# Неповторимый В.И.Кандрор

**DOI:** 10.14341/probl12753

**Published:** 2021-04-21

**Authors:** Е. А. Трошина

**Affiliations:** Национальный медицинский исследовательский центр эндокринологии

## Abstract

Не бывает незаменимых, но есть неповторимые… Это про Вилена Иосифовича Кандрора,  человека, ученого, профессионала, нашего дорогого  коллегу, с которым мы простились в этом году.

**Figure:**
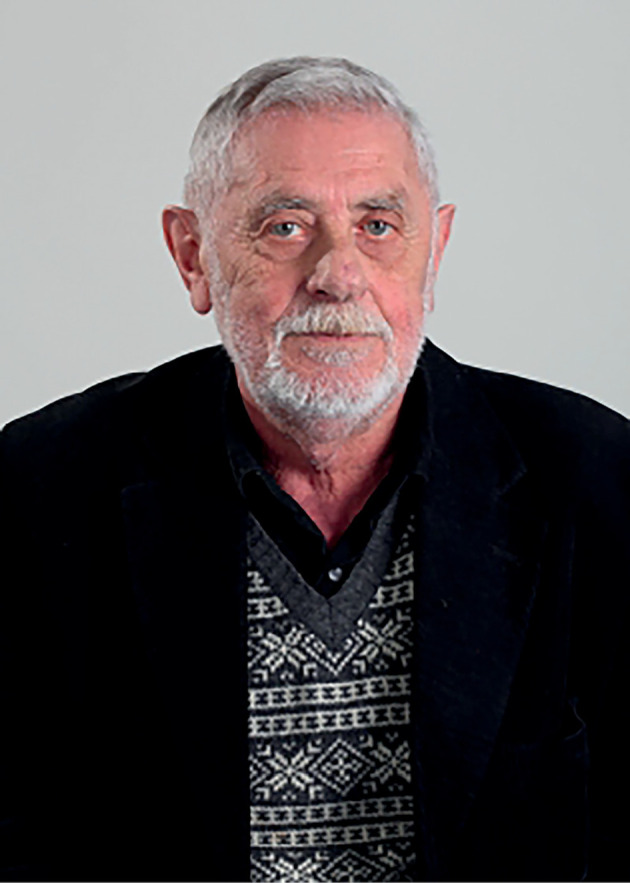


Не бывает незаменимых, но есть неповторимые…Это про Вилена Иосифовича Кандрора, человека, ученого, профессионала, нашего дорогого коллегу, с которым мы простились в этом году.

Возможно ли быть Интеллигентом без высочайшего интеллекта, доброжелательности, чувства юмора? Нет, конечно. Профессор Кандрор навсегда останется примером именно Интеллигента. Обладая энциклопедическими знаниями, Вилен Иосифович не «накапливал и хранил» их, а анализировал, развивал, приумножал и, что очень важно, — щедро ими делился. Фундаментальные исследования в области аутоиммунной патологии щитовидной железы профессора Кандрора золотыми буквами вписаны в историю эндокринологии. Книги, адаптированные переводы, лекции — нам, кто пришел в ЭНЦ в середине 90-х годов, очень повезло, что мы учились у него. До сих пор записи лекций сохранились, причем их содержание поражает своей актуальностью, несмотря на прошедшие годы. Просто Вилен Иосифович обладал одним из важнейших свойств ученого — научной прозорливостью, поэтому всегда был современен, а высказанные идеи и сейчас остаются основой для будущих изысканий.

Он был абсолютно доступен для разговора, обсуждения, дискуссии. Если нужен был совет, он давал его, причем не формально, а именно по делу. Запомнились блистательные выступления профессора Кандрора на ученых советах. Всегда — просто о сложном, никогда не обижая оппонента («Спасибо, если я правильно понимаю…»), но удивительным образом расставляя вcе точки над i таким образом, что становилось очевидным, кто «со щитом», а кто, увы…

Получить от профессора Кандрора одобрение идеи, статьи, книги — это можно было приравнять к получению знака качества. Не скрою, и я была в числе тех, кто пытался заслужить его уважение профессионала, сделать это было непросто. Я очень дорожу предисловием к одной из своих книг, которое было написано Виленом Иосифовичем, причем по его инициативе. Ему было дело до всего — от формулировок до сути, от аннотации аспиранта до монографии ученого. Вилен Иосифович не был равнодушным человеком, мы ощущали это на себе. Чувство юмора профессора — тема особая. И добрая ирония, и уместно рассказанный анекдот, умение посмеяться над собой — тоже пример блистательного умения жить и интересоваться жизнью во всех ее проявлениях. Он был красивым человеком. Тот случай, где форма и содержание — едины.

Важно, что Вилен Иосифович находил время (и делал это с удовольствием!) для общения с молодежью. Приведу ниже отрывок из его интервью, которое Вилен Иосифович дал студентке Первого меда – вуза, гимн которого переписать невозможно.

Каждый год приносит что-то новое и уникальное в историю первого МГМУ им. Сеченова. Многое меняется, но выпускники и студенты неизменно поют «Уходят в даль московских улиц ленты…», когда речь заходит о гимне их alma mater. К сожалению, припев знают все, а автора этих строк могут назвать единицы. Вилен Иосифович Кандрор с улыбкой вспоминает процесс создания гимна и делится им с нами, позволяя окунуться в студенческую жизнь 50-х годов.

Из интервью

## КАК ВОЗНИКЛА ИДЕЯ СОЗДАНИЯ ГИМНА?

Это была идея... не гимна. Тогда шел 1951 год, я был на 3-м или 4-м курсе. Дело в том, что в те годы у нас была очень бурная литературно-эстрадная жизнь, скажем так. Вы знаете, наше время — это было время создания КВН, а до него на нашем курсе устраивались эстрадные представления, и нужна была заключительная песня. «Входная» песенка была, номера были, все было – а финальной еще не было. И в качестве точки нашего выступления мы как раз и написали тогдашнюю финалочку и нынешний гимн с моим другом Сашей Шабалиным. Мы, значит, с ним писали песню на лекции, естественно. Саша что-то свое карябал, я — свое, а потом мы представили наше творение на суд организатору капустников, и он сказал: «Знаете что, пусть слова первого будут куплетом, а второго — припевом». Так, Сашка написал: «Вспомни, друг, как в ночь перед экзаменом...», а я написал: «Уходят в даль московских улиц ленты...», потом мы с ним сочинили последний куплет — кстати, все почему-то поют совершенно неправильно, там было так: «Скоро жизнь откроет двери ИМ», а поют «НАМ», это неверно. Значит, это написали, но песенка получилась какая-то коротенькая, нужно было написать промежуточный куплет, что я и сделал. И когда она стала исполняться, мы не думали, что это будет гимн. Музыку придумала наша же сокурсница Неля Осипова, даже, на самом деле, не придумала, она хорошо бренчала на фоно, а в то время была модной песня «Море в Гаграх, о пальмы в Гаграх», и что-то Неля с ней намудрила и предоставила мелодию к нашим стихам. Вот и вся история создания. 

## КАК ПОЛУЧИЛОСЬ ТАК, ЧТО ВЫ РЕШИЛИ ПОСТУПАТЬ В ПЕРВЫЙ МЕДИЦИНСКИЙ?

Что может хотеть восемнадцатилетний пацан, что он знает вообще? Дело в том, что мой отец, хоть он был и не медиком, а биологом, занимался акклиматизацией человека, что было все-таки близко к медицине. Как раз в то время, когда нужно было принимать решение, я написал шуточные строки: «Осознав в 10-м классе, что ждать медаль — напрасный труд, на поступленье был согласен в какой угодно институт, за исключеньем тех, однако, где торжествует Интеграл — он в математике собаку принципиально в рот не брал». Всем, вероятно, знакома такая философия — никаких гуманитарных вузов, потому что это точно не кусок хлеба, а специальность иметь надо, против технических я сам бунтовал. Поэтому, если не физика и математика, если не гуманитарное направление, то что остается? Естественно —научное.

## КАКАЯ КАФЕДРА СТАЛА ЛЮБИМОЙ?

Кафедра биохимии, я почти сразу записался в кружок. Там были достаточно тяжелые экспериментальные работы, некоторые из которых приходилось делать даже ночью. Все это я и мои товарищи находили очень увлекательным, так что в биохимическом корпусе мы почти жили.

## В ИТОГЕ ВЫ НЕ РАЗОЧАРОВАЛИСЬ В СВОЕМ ВЫБОРЕ?

Я никогда не работал врачом, но со студенческих лет любил биохимию. Правда, при кафедре меня не оставили и я поступил в институт имени Эрисмана, всю жизнь проработал в лаборатории. Так что, несмотря на то, что в дипломе у меня «лечебное дело», я сразу стал экспериментатором и никогда не работал с людьми.

## ЯВЛЯЕТЕСЬ ЛИ ВЫ АВТОРОМ УЧЕБНИКОВ И НАУЧНЫХ РАБОТ?

Компилятивные вещи писал, во всех изданиях Эндокринологического научного центра есть мои главы, но это несерьезно. У меня есть 4 монографии, что более существенно. Я всю жизнь занимался тиреотоксикозом, и в частности миокардом при тиреотоксикозе, у меня есть солидная монография, которая была моей докторской диссертацией — «Тиреотоксическое сердце». Учебные пособия также писал, однако очень много времени уделял переводам, смею утверждать, что большая часть литературы по эндокринологии, переведенной на русский язык, — это мое. Сейчас стараюсь следить за современными публикациями в научной и образовательной сферах, но, конечно, уже меньше, чем раньше, и только по своей очень узкой специальности.

«Уходят в даль московских улиц ленты…», подчас очень далеко ведут эти пути, но почему-то кажется, что где-то там, перед самым поворотом, Вилен Иосифович обернется и скажет слова, которые он адресовал молодежи, отвечая на последний вопрос интервью: «Что бы Вы пожелали нынешним студентам?»

Профессор Кандрор В.И.: «Ребята, живите весело — мой вам совет. Со временем убеждаешься, что жизнь очень стремительно проходит. Могу признаться, что первое ощущение того, как это быстро, пришло ко мне довольно рано — лет в сорок. Я вдруг подумал: “Почему меня не предупредили, что это все так быстро? Вот-вот это было — гуляли, веселились, песни пели, и вдруг уже ничего такого нет…”. Поэтому живите весело, а значит — интересно!»

